# A new player in an old process: ftsZ3 is necessary for chloroplast division in *Physcomitrium patens*

**DOI:** 10.1093/plcell/koag056

**Published:** 2026-03-04

**Authors:** Vanessica Jawahir

**Affiliations:** Assistant Features Editor, The Plant Cell, American Society of Plant Biologists; Donald Danforth Plant Science Center, Saint Louis, MO, United States

When my adventurous eating gets the better of me, I hope my indigestion only causes discomfort. The only positive outcome I expect is a lesson; I cannot fathom the result being a keystone event in the evolution of Plantae and life on Earth. But that is exactly what happened over a billion years ago when a protist engulfed a cyanobacterium but failed to digest its meal and allowed the cyanobacterium to persist as an endosymbiont (Gould et al. 2008; Sagan 1967). This singular event gave rise to chloroplasts in red and green algae and in glaucophytes. Secondary endosymbiotic events between algae and eukaryotes allowed plastids to spread laterally between eukaryotic lineages. These endosymbiotic events gave rise to all phototrophic eukaryotes (Rodríguez-Ezpeleta and Philippe 2006).

After eons of evolution of the endosymbiont and host, plastids vary in color, size, number, molecular composition, and even function (Choi et al. 2021). Despite losing autonomy, chloroplasts still exhibit some bacteria-like behavior. For example, chloroplasts, like bacteria, divide through binary fission. A ring-like protein complex forms on both the inner and outer chloroplast membranes followed by coordinated constriction of the ring that pinches the chloroplast into two.

A robust working model of chloroplast division has been established in Arabidopsis but cannot be applied universally to other plant species. In mosses and algae, several Arabidopsis division components are absent, whereas others have additional isoforms (Okazaki et al. 2010). Jintao Lang and colleagues (Lang et al. 2026) are filling in knowledge gaps associated with chloroplast division in Streptophyta and developed a model of chloroplast division in *Physcomitrium patens* by performing a large-scale characterization of the function, localization, and protein-protein interactions of chloroplast division components.

Lang et al. (2026) uncovered a novel mechanism in which Filamenting temperature-sensitive Z 3 (FtsZ3) temporospatially regulates chloroplast division. FtsZ has evolved into 3 distinct families in algae and plants. Most plants possess 2 isoforms, FtsZ1 and FtsZ2, while FtsZ3 is exclusive to algae, bryophytes, and lycophytes, but its role in chloroplast division was poorly understood until now.

FtsZ proteins have a conserved GTPase region and a C-terminal peptide (CTP) region that interact with other division proteins and tethers FtsZ to the membrane. These regions were perturbed using CRISPR/Cas9 in *P. patens* to generate a suite of mutants. Frame shift mutants *ftsZ3-fs* resulted in severe chloroplast division phenotypes with long filamentous undivided chloroplasts, demonstrating that FtsZ3 functions in chloroplast division. FtsZ3 mutants lacking the CTP *ftsZ3_ΔC-Ter_* exhibited enlarged, dumbbell-shaped chloroplasts, while disruption of the GTPase domain, *ftsZ3*_PV242HΔ_, resulted in a milder phenotype of elongated filamentous chloroplasts that are smaller than those observed in *ftsZ3-fs* but are more severe than those of *ftsZ3_ΔC-Ter_*. The distinct phenotypes arising from *ftsZ3* alleles demonstrate that FtsZ3 is necessary for proper chloroplast division and that the GTPase and CTP domains have different roles.

CRISPR/Cas9 knock-in technology was used to generate an endogenous GFP-tagged FtsZ3 enabling the localization of the wild-type protein and *ftsZ3_ΔC-Ter_*. FtsZ3-GFP localized to a ring-like structure in both early and late stages of chloroplast division, demonstrating that FtsZ3 participates throughout the duration of the division process. FtsZ3_ΔC-Ter_-GFP was still detected in the division ring, although the chloroplasts were unable to complete fission; this indicates that the CTP is not necessary for FtsZ3 localization at the division ring. FtsZ3_PV242HΔ_ were localized to speckles and failed to consistently localize to the division ring suggesting. The difference in localization of mutants disrupted for the GTPase and for C-terminal peptide domains supports the proposal that the GTPase domain is necessary for FtsZ3 recruitment to the division ring.

Turbo ID-based proximity labeling proteomics showed that FtsZ3 interacts with the known CDM components FtsZ1-1, FtsZ1-2, FtsZ2-1, and Accumulation and Replication of Chloroplasts 6 (ARC6). A combination of yeast 2-hybrid experiments and immunofluorescence was used to dissect interactions between each domain of FtsZ3 and its partner proteins. Yeast 2-hybrid assays validated that the CTP domain of FtsZ3 interacts with the stromal regions of ARC6, while the GTPase domain interacts with FtsZ2.

The localization and interaction experiments support a model in which FtsZ3 interacts with FtsZ2 at their GTPase domains to initiate assembly of the internal division ring at the early stages of chloroplast division. The CTP of FtsZ3 interacts with ARC6 to facilitate constriction of the ring to finalize division (Fig. 1). Lang et al. discovered a novel mechanism of chloroplast division dependent on the different roles of FtsZ3 throughout the chloroplast division process. FtsZ3 is critical for chloroplast division in moss but absent in land-plants, which serves as a reminder of the often underappreciated complexity of early diverging land plants and the moss-specific evolution of chloroplast division machinery. This study generated a working model of chloroplast division and CDM components in *P. patens* that provides a crucial snapshot of the diversity in chloroplast division machinery and their function.

**Figure 1:**
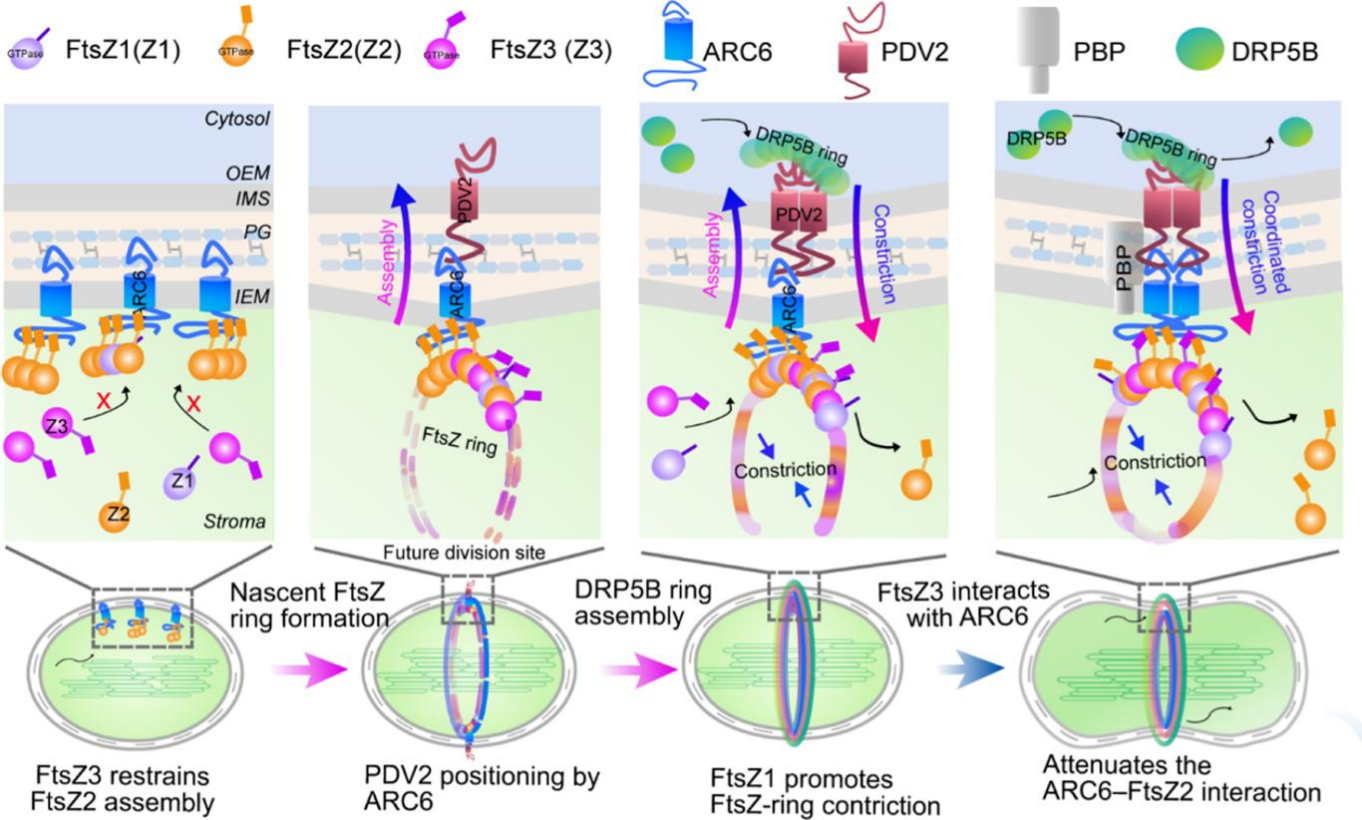
Model of chloroplast division in *P. patens*. FtsZ2 interacts with ARC6 to form the scaffold for ring assembly. FtsZ3 interacts with FtsZ2 through their GTPase domains to form the division ring. Proteins are recruited to assemble division ring on the outer membrane. FtsZ3 is incorporated more into the ring and interacts with ARC6 to constrict the ring to complete division. Adapted from Lang et al. (2026) Figure 6.

## Recent related articles in *The Plant Cell*:

- Liu et al. (2022) described how FtsZ1 has weak membrane-binding activity because of an amphiphilic motif at its C terminus, which contributes to proper targeting to facilitate chloroplast division (Liu et al 2022).
- Chen et al. (2019) reported that Arabidopsis possesses 2 pools of ARC3. The stromal pool inhibits Z-ring assembly at nondivision sites, while the other pool is localized to the division site (Chen et al. 2019).

## Data Availability

No new data were generated in the preparation of this manuscript.
